# Pirfenidone Suppresses Liver Fibrosis Through Inhibition of TGF-β-Associated Lipid Metabolic Remodeling in Hepatic Stellate Cells

**DOI:** 10.3390/ijms27094061

**Published:** 2026-04-30

**Authors:** Yuelu Lan, Sijia Li, Shuangli Zhu, Can Pan, Kai Fu, Xueping Wang, Liwu Fu, Fang Wang

**Affiliations:** State Key Laboratory of Oncology in South China, Guangdong Provincial Clinical Research Center for Cancer, Sun Yat-sen University Cancer Center, Guangzhou 510060, China; lanyl@sysucc.org.cn (Y.L.); lisj1@sysucc.org.cn (S.L.); zhusl@sysucc.org.cn (S.Z.); pancan@sysucc.org.cn (C.P.); fukai@sysucc.org.cn (K.F.); wangxuep@sysucc.org.cn (X.W.)

**Keywords:** hepatic stellate cell (HSC), transforming growth factor-β (TGF-β), lipogenesis/lipid metabolism, pirfenidone, liver fibrosis, SREBP1, FASN

## Abstract

Chronic liver injury is characterized by sustained activation of transforming growth factor-β (TGF-β) signaling within the fibrotic microenvironment, yet the contribution of TGF-β-associated metabolic remodeling to hepatic stellate cell (HSC) activation remains incompletely understood. Here, we investigated whether TGF-β signaling is associated with lipid metabolic remodeling in HSCs and whether pirfenidone (PFD) interferes with this process. We found that TGF-β1 was spatially associated with lipid accumulation in fibrotic liver tissue and that TGF-β1/2 promoted HSC proliferation. In vitro, TGF-β1/2 coordinately upregulated sterol regulatory element-binding protein 1 (SREBP1) and fatty acid synthase (FASN), accompanied by increased intracellular lipid accumulation and enhanced oleic acid (OA)-associated lipid responses. Low-dose OA further activated AKT/ERK/p70 S6K signaling in HSCs, whereas PFD attenuated these signaling events. In parallel, PFD suppressed TGF-β-associated lipid accumulation in vitro, reduced SREBP1/FASN expression in activated HSC-rich regions in vivo, and alleviated CCl_4_-induced liver fibrosis. Together, these findings support a model in which TGF-β-associated lipogenic remodeling contributes to HSC activation and suggest that interference with this metabolic state may represent one component of the antifibrotic action of pirfenidone.

## 1. Introduction

Liver fibrosis is a common pathological consequence of chronic liver injury caused by viral hepatitis, alcohol abuse, and non-alcoholic fatty liver disease (NAFLD), and represents a critical intermediate stage preceding cirrhosis and hepatocellular carcinoma [1]. NAFLD is the most common chronic liver disease worldwide, with its disease spectrum encompassing stages ranging from simple steatosis, NASH, fibrosis, and cirrhosis to hepatocellular carcinoma [2]. Despite its diverse etiologies, fibrogenesis is driven by a shared cellular program centered on the activation of hepatic stellate cells (HSCs) [3,4]. Upon liver injury, quiescent, lipid-storing HSCs transdifferentiate into proliferative, myofibroblast-like cells that secrete excessive extracellular matrix, thereby disrupting normal liver architecture and function [5,6].Transforming growth factor-β (TGF-β) signaling is widely recognized as a master regulator of HSC activation and fibrogenesis. Canonical TGF-β pathways promote the transcription of profibrotic genes, including collagen and α-smooth muscle actin, and sustain myofibroblastic phenotypes [7,8,9]. TGF-β signaling has been shown to be upregulated as early as the simple steatosis stage, suggesting that TGF-β-driven lipid metabolism remodeling may already be initiated during the early metabolic dysfunction phase of NAFLD, even before significant inflammatory infiltration occurs [10,11]. Beyond its classical role in extracellular matrix production, accumulating evidence indicates that TGF-β signaling also exerts broad effects on cellular metabolism [12,13,14]. In cancer and fibrotic diseases, TGF-β has been shown to remodel glucose and lipid metabolism in ways that support cellular growth, survival, and phenotypic transition [15,16]. However, whether TGF-β-associated metabolic remodeling directly contributes to HSC activation, and particularly whether lipid metabolic remodeling represents a functionally relevant component of this process, remains incompletely understood.

Lipid metabolism is a defining feature of HSC biology. Quiescent HSCs are characterized by abundant cytoplasmic lipid droplets enriched in retinoids, whereas HSC activation is accompanied by marked lipid droplet remodeling and broader metabolic adaptation [5,6]. Emerging evidence suggests that these changes are not merely passive consequences of phenotypic transition, but may actively support HSC proliferation and fibrogenic signaling through altered fatty acid synthesis, uptake, and utilization. In this context, key lipogenic regulators such as sterol regulatory element-binding protein 1 (SREBP1) and its downstream target fatty acid synthase (FASN) have attracted increasing attention in cancer and metabolic liver disease [17,18,19]. Nevertheless, the upstream signals that engage this lipogenic machinery in HSCs, and its functional relevance to fibrogenesis, remain to be fully elucidated.

Pirfenidone (PFD) is an orally available antifibrotic agent approved for the treatment of idiopathic pulmonary fibrosis and has demonstrated therapeutic potential in experimental liver fibrosis [20]. Its reported mechanisms include anti-inflammatory, antioxidant, and TGF-β-modulatory effects [21,22]. However, whether pirfenidone exerts part of its antifibrotic activity through modulation of metabolic remodeling in HSCs remains unclear. Given the growing recognition of metabolism as an active driver of fibrotic disease, clarifying whether a clinically relevant antifibrotic drug can interfere with this metabolic component of HSC activation may provide new insight into the pharmacological actions of pirfenidone and broaden its therapeutic relevance.

In this study, we tested the hypothesis that TGF-β signaling promotes HSC activation in association with lipid metabolic remodeling and that pirfenidone counteracts liver fibrosis, at least in part, by suppressing this process. We found that TGF-β1 is spatially associated with lipid accumulation in fibrotic liver tissue and that TGF-β1/2 promote HSC proliferation together with coordinated upregulation of SREBP1 and FASN. These changes are accompanied by increased intracellular lipid accumulation, enhanced oleic acid (OA)-associated lipid responses, and activation of AKT/ERK/p70 S6K signaling. Importantly, pirfenidone suppresses multiple nodes of this metabolic-signaling circuit in vitro and in vivo and attenuates CCl_4_-induced liver fibrosis. Together, these findings support a model in which TGF-β-associated lipogenic remodeling contributes to HSC activation and suggest that interference with this pathway may represent one component of pirfenidone’s antifibrotic action.

## 2. Results

To determine whether TGF-β-driven lipid metabolic remodeling contributes to HSC activation and whether pirfenidone interferes with this process, we examined the pathway at four levels: pathological association in vivo, activation of lipogenic programs in vitro, downstream proliferative signaling, and pharmacological validation in fibrotic liver.

### 2.1. TGF-β1 Is Spatially Associated with Lipid Accumulation in Fibrotic Liver and Promotes HSC Proliferation

**Figure 1:**
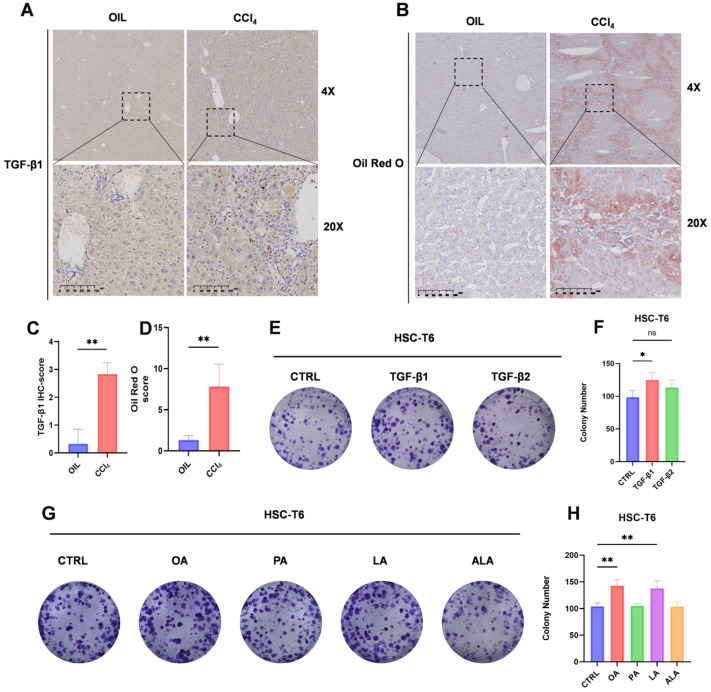
TGF-β1 is associated with hepatic lipid accumulation and promotes HSC proliferation. (**A**) Images of liver sections from control and CCl_4_-treated mice stained for TGF-β1. (**B**) Oil Red O staining of liver sections from control and CCl_4_-treated mice. (**C**,**D**) Quantification of TGF-β1-positive and Oil Red O-positive areas. (**E**) Colony formation images of HSC-T6 cells treated with TGF-β1 or TGF-β2 (10 ng/mL). (**F**) Quantification of colony numbers from three independent experiments (as exemplified in (**E**)). (**G**) Colony formation images of HSC-T6 cells treated with the indicated fatty acids (25 μM). (**H**) Quantification of colony numbers from three independent experiments (as exemplified in (**G**)). Quantification was performed from randomly selected fields under blinded conditions. Data are presented as the mean ± SD. (**C**,**D**) Unpaired Student’s *t*-test. (**F**,**H**) One-way ANOVA with Tukey’s HSD post hoc test. Significant changes are indicated as follows: *, *p* < 0.05; **, *p* < 0.01; ns indicates not significant (*p* ≥ 0.05), compared to the control group.

It has been reported that TGF-β signaling becomes activated at the simple steatosis stage, and functions during the early stages of a broad spectrum of diseases [10,11]. We first investigated whether TGF-β signaling is associated with abnormal lipid deposition in fibrotic liver tissue. In a carbon tetrachloride (CCl_4_)-induced mouse model of liver fibrosis, TGF-β1 immunoreactivity exhibited marked spatial overlap with Oil Red O-positive lipid signals in nonparenchymal regions of the liver, suggesting a close association between TGF-β activation and local lipid remodeling under fibrotic conditions (Figure 1A–D).

To evaluate the direct effects of TGF-β signaling on hepatic stellate cell behavior, HSC-T6 cells were treated with TGF-β1 or TGF-β2. Both isoforms significantly increased colony formation, indicating enhanced proliferative capacity (Figure 1E,F). We next screened several exogenous fatty acids for their effects on HSC proliferation. Among the fatty acids tested, OA and linoleic acid (LA) promoted colony formation, with OA producing the most pronounced effect (Figure 1G,H). Based on these findings, TGF-β1, TGF-β2, and OA were selected for subsequent experiments. Together, these data suggest that TGF-β signaling and lipid-related cues converge on a pro-proliferative phenotype in HSCs and prompted us to investigate whether TGF-β directly reprograms lipid metabolism in these cells. Each experiment was independently repeated three times, and data are presented as the mean ± SD from three independent biological replicates.

### 2.2. TGF-β1/2 Coordinately Upregulate SREBP1 and FASN in Hepatic Stellate Cells

**Figure 2:**
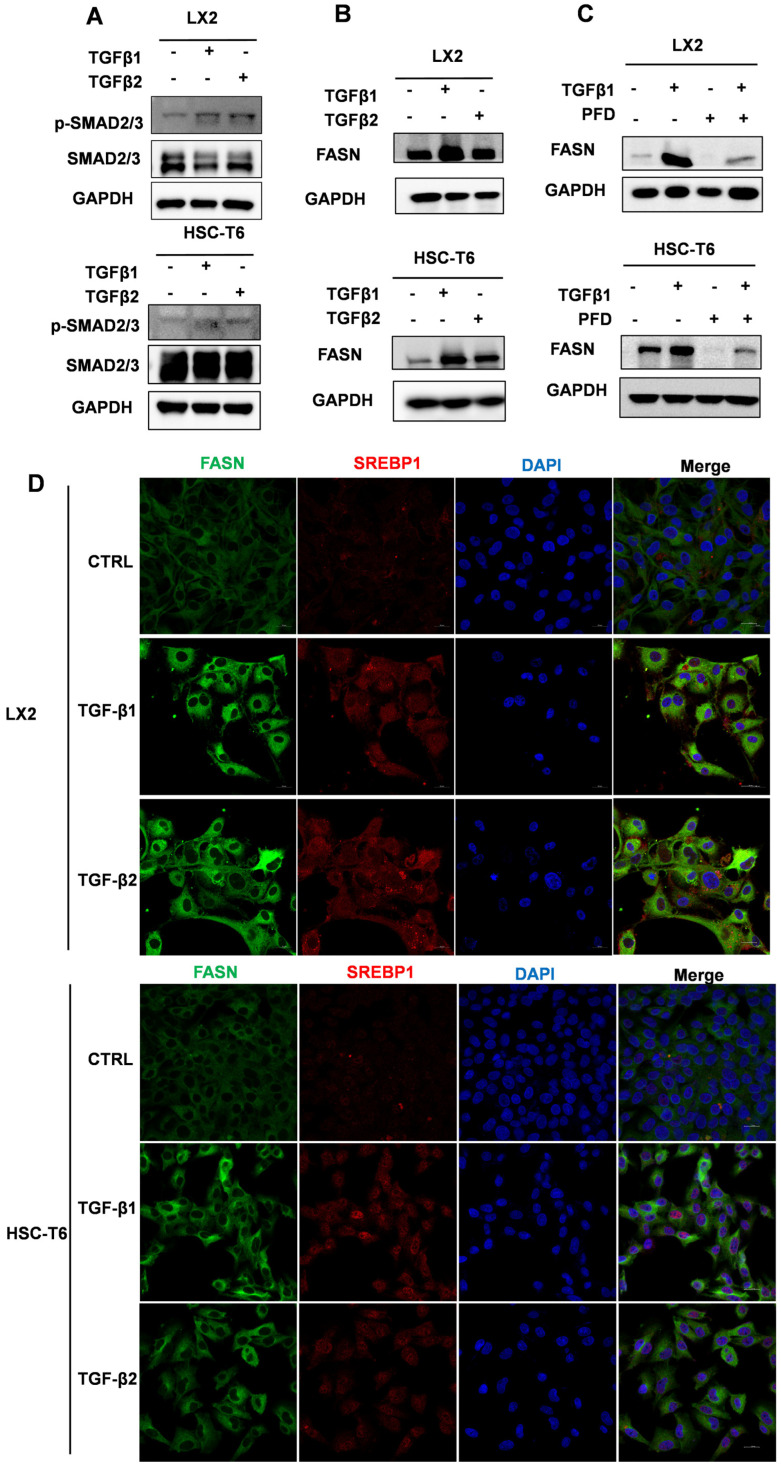
TGF-β1/2 coordinately upregulate SREBP1 and FASN in HSCs. (**A**) Western blot detection of phospho-SMAD2/3 and total SMAD2/3 in LX2 and HSC-T6 cells treated with TGF-β1 or TGF-β2 (10 ng/mL, 48 h). (**B**,**C**) Western blot detection of FASN expression in LX2 and HSC-T6 cells following TGF-β1 or TGF-β2 (10 ng/mL, 48 h) stimulation with or without PFD (250 μg/mL). (**D**) Immunofluorescence images of LX2 and HSC-T6 cells stained for FASN (green), SREBP1 (red), and nuclei (DAPI, blue) after treatment with TGF-β1 or TGF-β2 (10 ng/mL, 48 h).

To define the molecular basis by which TGF-β signaling may influence lipid metabolism in HSCs, we first examined canonical pathway activation and the expression of lipogenesis-related regulators. Western blot analysis confirmed that TGF-β1 and TGF-β2 robustly increased SMAD2/3 phosphorylation in both LX2 and HSC-T6 cells, indicating effective pathway activation (Figure 2A). Under the same treatment conditions, FASN protein expression was markedly upregulated in both cell lines (Figure 2B). PFD suppresses TGF-β1–induced FASN upregulation in vitro (Figure 2C). Cell viability under different pirfenidone concentrations was assessed by MTT assay in LX2 and HSC-T6 cells. At 250 μg/mL for 48 h, viability remained above 80% in both cell lines (Figure S1).

Because SREBP1 is a major transcriptional regulator of lipogenic gene expression, we next examined whether TGF-β stimulation was accompanied by coordinated changes in SREBP1 and FASN [17,18,19]. Immunofluorescence staining showed that both TGF-β1 and TGF-β2 increased SREBP1 and FASN signals in LX2 and HSC-T6 cells, with substantial overlap between the two proteins (Figure 2D). These findings indicate that TGF-β signaling is accompanied by activation of a lipogenic program characterized by coordinated upregulation of SREBP1 and FASN in HSCs. We therefore next asked whether this molecular response is associated with measurable changes in intracellular lipid accumulation.

### 2.3. TGF-β Signaling Enhances Intracellular Lipid Accumulation in Hepatic Stellate Cells, Which Is Suppressed by Pirfenidone

**Figure 3:**
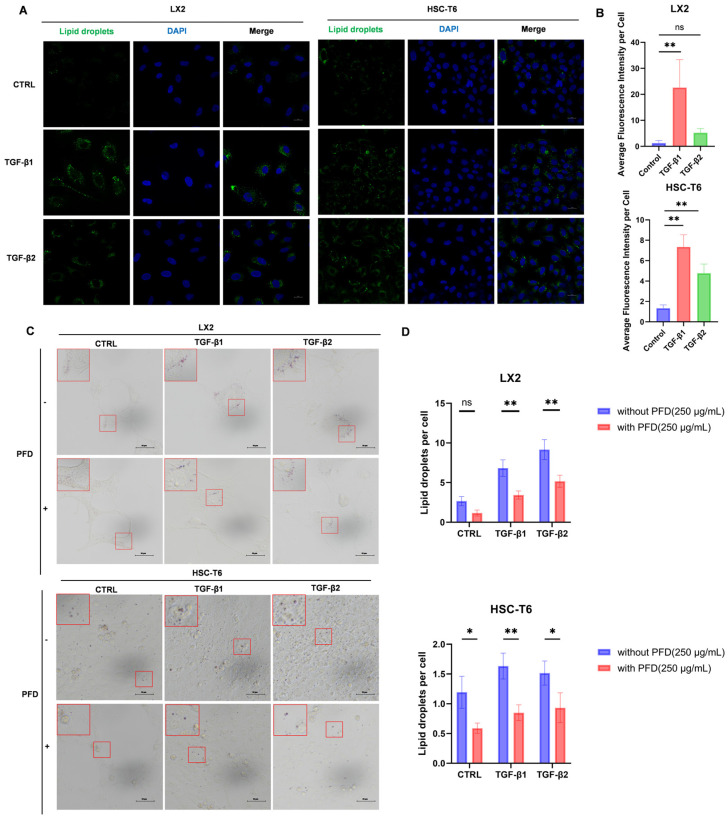
TGF-β signaling enhances intracellular lipid accumulation in HSCs, which is suppressed by pirfenidone. (**A**) BODIPY 493/503 staining images of LX2 and HSC-T6 cells after serum starvation followed by TGF-β1 or TGF-β2 (10 ng/mL) treatment for 48 h. (**B**) Quantification of mean BODIPY fluorescence intensity in LX2 and HSC-T6 cells. (**C**) Oil Red O staining images of LX2 and HSC-T6 cells treated with TGF-β1 or TGF-β2 (10 ng/mL, 48 h) in the presence or absence of PFD (250 μg/mL) after serum starvation. The red square indicates the region shown at higher magnification in the upper left corner of the same subfigure. (**D**) Quantification of Oil Red O-stained lipid droplets per cell in LX2 and HSC-T6 cells. Quantification was performed from randomly selected fields under blinded conditions. Data are presented as the mean ± SD from three independent biological replicates. Significance was determined by one-way ANOVA with Tukey’s HSD post hoc test. Two-way ANOVA with Šídák’s post hoc test was used for comparisons between PFD-treated and untreated groups. *, *p* < 0.05; **, *p* < 0.01; ns indicates not significant (*p* ≥ 0.05).

To determine whether TGF-β-driven upregulation of lipogenic regulators is accompanied by a corresponding lipid phenotype, LX2 and HSC-T6 cells were serum-starved for 24 h to reduce basal intracellular lipid stores and then stimulated with TGF-β1 or TGF-β2 for 48 h. Lipid droplets were visualized using BODIPY 493/503 staining. In both cell lines, TGF-β1 significantly increased intracellular neutral lipid fluorescence, whereas TGF-β2 showed a similar trend that did not reach statistical significance under the tested conditions (Figure 3A,B).

We next assessed whether PFD interferes with TGF-β-associated lipid accumulation. After serum starvation, cells were treated with TGF-β1 or TGF-β2 in the presence or absence of PFD for 48 h, followed by Oil Red O staining. Compared with TGF-β treatment alone, PFD co-treatment markedly reduced intracellular neutral lipid droplet content in both LX2 and HSC-T6 cells (Figure 3C,D). These results indicate that TGF-β signaling enhances intracellular lipid accumulation in HSCs and that PFD effectively suppresses this response. Because intracellular lipid accumulation may reflect not only endogenous lipogenic activity but also altered handling of extracellular fatty acids, we next examined the effect of TGF-β on OA-associated lipid accumulation.

### 2.4. TGF-β Signaling Enhances OA-Associated Intracellular Lipid Accumulation in HSCs, Which Is Inhibited by Pirfenidone

**Figure 4:**
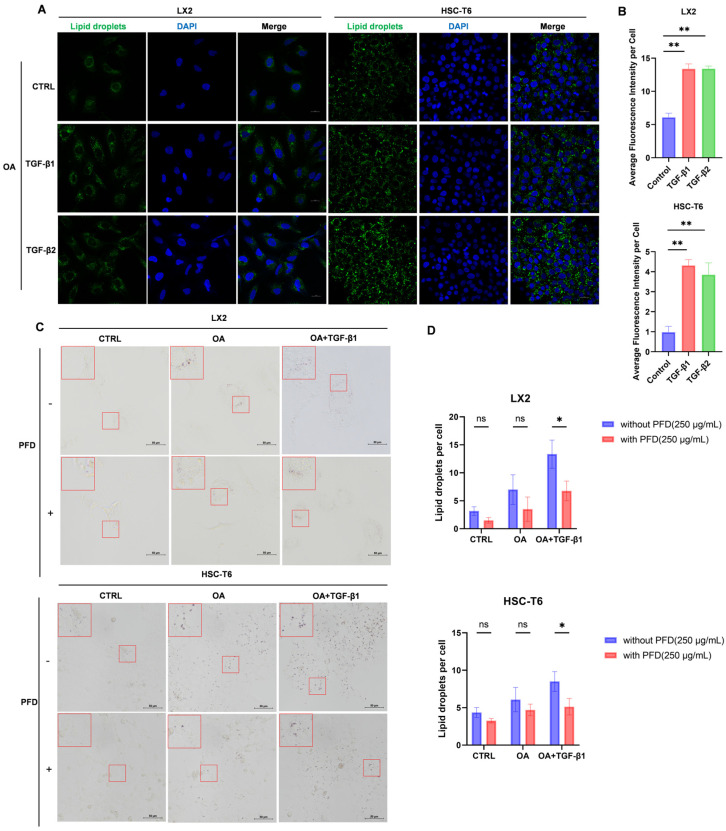
TGF-β signaling enhances OA-associated intracellular lipid accumulation in HSCs, which is inhibited by pirfenidone. (**A**) BODIPY 493/503 staining images of LX2 and HSC-T6 cells under serum starvation and pretreated with TGF-β1 or TGF-β2(10 ng/mL) for 24 h and then incubated with 200 μM OA for the final 4 h. (**B**) Quantification of OA-associated intracellular lipid accumulation in LX2 and HSC-T6 cells. (**C**) Oil Red O staining images of cells under serum starvation ± PFD (250 μg/mL) for 24 h. Groups: control; OA (200 μM, final 4 h); TGF-β1 (10 ng/mL, 24 h) + OA (200 μM, final 4 h). The red square indicates the region shown at higher magnification in the upper left corner of the same subfigure (**D**) Quantification of Oil Red O-stained lipid droplets per cell in LX2 and HSC-T6 cells. Quantification was performed from randomly selected fields under blinded conditions. Data are presented as the mean ± SD from three independent biological replicates. Significance was determined by one-way ANOVA with Tukey’s HSD post hoc test. Two-way ANOVA with Šídák’s post hoc test was used for comparisons between PFD-treated and untreated groups. *, *p* < 0.05; **, *p* < 0.01; ns indicates not significant (*p* ≥ 0.05).

To evaluate whether TGF-β signaling alters the cellular handling of exogenous fatty acids, we performed OA-associated lipid accumulation assays in LX2 and HSC-T6 cells. Cells were serum-starved and pretreated with TGF-β1 or TGF-β2 for 24 h, followed by incubation in complete medium containing OA for an additional 4 h in the continued presence of the corresponding TGF-β stimulus. BODIPY 493/503 staining showed that both TGF-β1 and TGF-β2 significantly increased OA-associated intracellular neutral lipid fluorescence in both cell lines (Figure 4A,B), consistent with enhanced utilization or uptake of exogenous OA.

We next examined whether PFD modifies this response. Under OA-containing conditions, TGF-β1 markedly increased intracellular lipid accumulation, whereas co-treatment with PFD substantially attenuated this effect (Figure 4C,D). These observations suggest that TGF-β signaling not only promotes a lipogenic intracellular state but also enhances the cellular response to extracellular OA, and that both processes are sensitive to PFD. Since low-dose OA promoted HSC proliferation in our initial screening, we next investigated whether OA activates proliferation-related signaling pathways in HSCs.

### 2.5. Low-Dose OA Activates AKT/ERK/p70 S6K Signaling in HSCs, and This Response Is Attenuated by Pirfenidone

**Figure 5:**
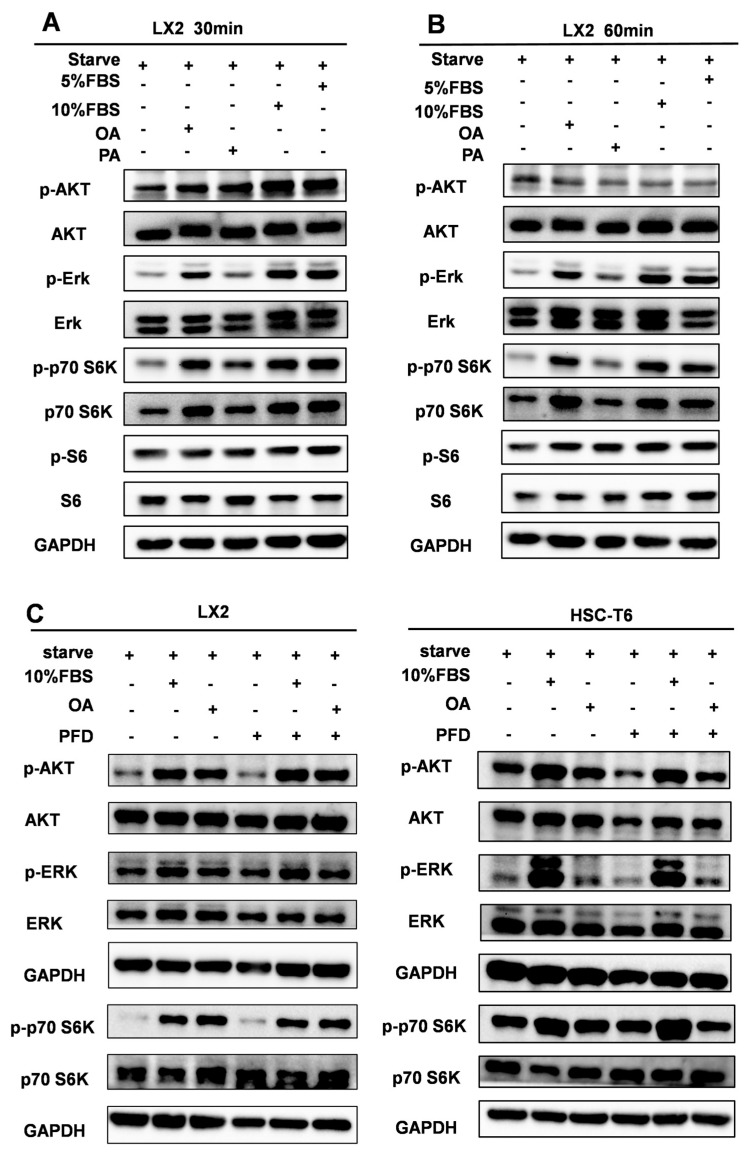
Low-dose OA activates AKT/ERK/p70 S6K signaling in HSCs, and this response is attenuated by pirfenidone. (**A**,**B**) Western blot detection of phospho-AKT, AKT, phospho-ERK, ERK and phospho-p70 S6K, p70 S6K in LX2 cells treated with OA or PA (25 μM) for 30 or 60 min, with 5% and 10% FBS as positive controls. (**C**) Western blot detection of phospho-AKT, AKT, phospho-ERK, ERK and phospho-p70 S6K, p70 S6K in LX2 and HSC-T6 cells treated with OA (25 μM) in the presence or absence of PFD (250 μg/mL) for 30 min.

Because OA enhanced HSC proliferation at low concentration, we sought to identify signaling pathways potentially linking lipid availability to proliferative responses. LX2 cells were serum-starved and then stimulated with OA (25 μM) or palmitic acid (PA) for 30 or 60 min, with 5% or 10% FBS used as positive controls. Western blot analysis showed that OA induced a more evident increase in the phosphorylation of AKT, ERK, and p70 S6K than PA, particularly at 30 min (Figure 5A,B). Based on this result, 25 μM OA for 30 min was selected for subsequent experiments.

To assess the effect of PFD on OA-responsive signaling, LX2 and HSC-T6 cells were serum-starved in the presence or absence of PFD and then stimulated with OA for 30 min. OA treatment markedly increased phosphorylation of AKT, ERK, and p70 S6K in both cell lines, whereas co-treatment with PFD substantially suppressed this response (Figure 5C). These data indicate that low-dose OA activates proliferation-associated signaling pathways in HSCs and that PFD attenuates this OA-responsive signaling output. Collectively, the in vitro findings support a model in which TGF-β-associated lipid remodeling is linked to enhanced proliferative signaling in HSCs. We next examined whether this pathway is also suppressed by PFD in vivo.

### 2.6. Pirfenidone Suppresses SREBP1/FASN Signaling in Activated HSC-Rich Regions In Vivo

**Figure 6:**
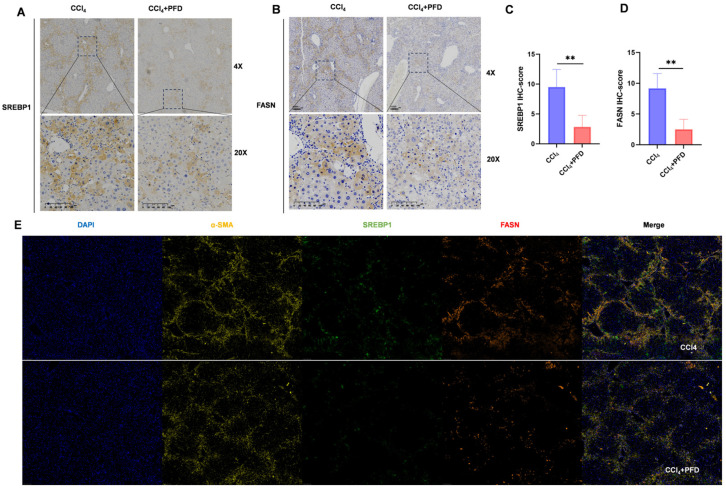
Pirfenidone suppresses SREBP1/FASN signaling in activated HSC-rich regions in vivo. (**A**,**B**) Immunohistochemical staining of SREBP1 and FASN in liver sections from control, CCl_4_, and CCl_4_ + PFD (400 mg/kg/day) groups. (**C**,**D**) Quantification of SREBP1 and FASN positive staining. (**E**) Multiplex immunofluorescence images of liver sections stained for α-SMA, SREBP1, and FASN. Quantification was performed from randomly selected fields under blinded conditions. Data are presented as the mean ± SD. Significant changes are indicated as follows: **, *p* < 0.01, compared to the control group (unpaired Student’s *t*-test).

To validate the in vitro findings in fibrotic liver tissue, we then analyzed liver tissues from CCl_4_-treated mice with or without PFD administration. Immunohistochemical staining demonstrated that both SREBP1 and FASN were markedly increased in fibrotic livers, whereas PFD treatment significantly reduced the expression of both proteins (Figure 6A–D). Notably, positive SREBP1 and FASN signals were enriched in nonparenchymal regions, particularly in areas with inflammatory infiltration.

To further define the cellular context of this response, multiplex immunofluorescence staining was performed for α-SMA, SREBP1, and FASN. In fibrotic liver sections, α-SMA-positive activated HSC-rich areas showed strong coincident signals for both SREBP1 and FASN. After PFD treatment, the α-SMA-positive area was reduced, and SREBP1/FASN signals within these regions were also markedly diminished (Figure 6E). These findings support the in vivo relevance of a lipogenic program in activated HSC-rich regions and indicate that PFD suppresses this program under fibrotic conditions.

### 2.7. Pirfenidone Attenuates CCl_4_-Induced Liver Fibrosis and HSC Activation In Vivo

**Figure 7:**
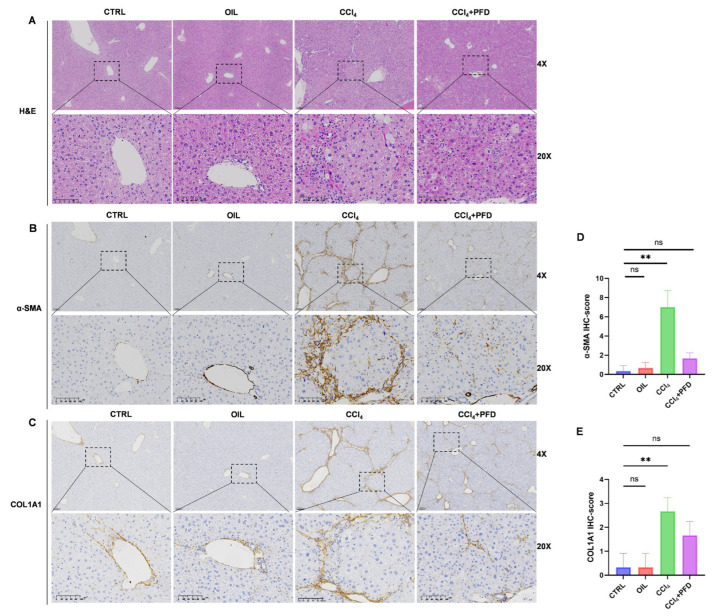
Pirfenidone attenuates CCl_4_-induced liver fibrosis and HSC activation in vivo. (**A**) H&E staining of liver sections from control, CCl_4_, and CCl_4_ + PFD (400 mg/kg/day) groups. (**B**) Immunohistochemical staining of α-SMA in liver sections from the indicated groups. (**C**) Immunohistochemical staining of COL1A1 in liver sections from the indicated groups. (**D**,**E**) Quantification of α-SMA and COL1A1 positive staining. Quantification was performed from randomly selected fields under blinded conditions. Data are presented as the mean ± SD. Significance was determined by one-way ANOVA with Tukey’s HSD post hoc test. ** *p* < 0.01; ns indicates not significant (*p* ≥ 0.05).

Finally, we evaluated the overall antifibrotic effect of PFD in the CCl_4_-induced liver fibrosis model, with PFD intervention administered concurrently during the early stage of TGF-β axis initiation in liver injury. Histological examination by H&E staining showed that PFD-treated mice exhibited reduced fibrotic septa formation and less inflammatory infiltration compared with the CCl_4_ model group (Figure 7A). In parallel, immunohistochemical staining demonstrated that PFD significantly decreased the expression of α-SMA, a marker of activated HSCs, and COL1A1, a major extracellular matrix component (Figure 7B–E).

Taken together, these in vivo data indicate that PFD attenuates liver fibrosis and HSC activation in parallel with suppression of the SREBP1/FASN-associated lipogenic program. Overall, our results support a model in which TGF-β signaling promotes a lipid-remodeled state in HSCs, characterized by coordinated upregulation of SREBP1/FASN, intracellular lipid accumulation, and enhanced OA-responsive proliferative signaling, whereas PFD interrupts multiple nodes of this circuit both in vitro and in vivo.

## 3. Discussion

Hepatic stellate cell activation is increasingly understood not only as a transcriptional and phenotypic transition but also as a process accompanied by profound metabolic adaptation. We show that TGF-β signaling is associated with a lipogenic remodeling program in HSCs and that pirfenidone interferes with this process.

These findings extend the view of TGF-β as a profibrotic cytokine that primarily drives extracellular matrix production. Our data suggest that, in HSCs, TGF-β is also associated with activation of a lipogenic program marked by coordinated upregulation of SREBP1 and FASN, linking profibrotic signaling to metabolic state transition. Given that SREBP1/FASN-dependent lipid synthesis supports growth and adaptation in other pathological contexts [23,24,25,26], our results support the idea that TGF-β-associated metabolic remodeling may contribute to sustaining an activated HSC phenotype rather than merely accompany it.

This study addresses both intracellular lipid accumulation and the cellular response to extracellular OA. As low-dose OA promoted HSC proliferation and activated AKT/ERK/p70 S6K signaling, extracellular fatty acids may provide more than passive metabolic substrates in activated HSCs. Instead, altered handling of fatty acids may reinforce proliferation-associated signaling outputs and thereby support fibrogenic activation. In this context, the lipid-remodeled state induced by TGF-β may serve as an interface through which metabolic inputs are coupled to growth-promoting signaling pathways.

Our data also add a metabolic dimension to the antifibrotic actions of PFD. PFD consistently suppressed several nodes of the TGF-β-associated metabolic program, including intracellular lipid accumulation, OA-induced phosphorylation of AKT/ERK/p70 S6K, and SREBP1/FASN expression in activated HSC-rich regions in vivo. These observations suggest that interference with metabolic adaptation in HSCs may contribute, at least in part, to the antifibrotic effects of PFD. Thus, beyond its established pharmacological properties, PFD may also function as a modulator of fibrosis-associated metabolic remodeling.

The present findings should also be interpreted in the broader context of metabolic reprogramming during HSC activation. Previous studies have shown that TGF-β signaling can influence glucose metabolism, mitochondrial dynamics, and other bioenergetic processes in fibroblastic cells and HSC-related models [27,28]. Together with those reports, our study supports the concept that TGF-β-driven fibrogenesis involves coordinated remodeling of multiple metabolic axes, including both glycolytic and lipid-associated pathways. From a translational perspective, this raises the possibility that metabolic nodes may represent therapeutically actionable vulnerabilities in liver fibrosis. In particular, the SREBP1/FASN-associated axis identified here may represent one component of a broader metabolism-fibrosis network that sustains HSC activation.

In the context of NAFLD, compelling evidence indicates that TGF-β signaling becomes upregulated as early as the simple steatosis stage, before the diagnostic criteria for NASH are met [10,11]. At this early phase, hepatocyte lipotoxicity and Kupffer cell activation can trigger TGF-β1 secretion, promoting HSC transdifferentiation and ECM deposition even in the absence of overt lobular inflammation [29,30]. This challenges the classical view that fibrogenesis is a late-stage phenomenon and supports the concept that therapeutic strategies targeting the TGF-β axis should be implemented early in fatty liver disease.

Several limitations of this study should be acknowledged. First, the CCl_4_-induced model does not fully recapitulate the metabolic features of human NAFLD, as it relies on direct hepatocyte necrosis rather than chronic lipid overload. Second, a formal prospective power analysis was not performed. Although post hoc power analysis supported the adequacy of the present sample size, a prospective sample size calculation was not performed and should be incorporated in future confirmatory studies. Third, parallel cell viability assays were not included to distinguish proliferation from survival effects in the colony formation experiments. Future studies will address these limitations using NAFLD-specific models and more rigorous experimental designs. Although our data support a model in which pirfenidone suppresses TGF-β-associated lipogenic remodeling in activated HSCs, the present study does not yet establish a strict linear causal hierarchy linking TGF-β signaling, SREBP1/FASN activation, and downstream proliferative signaling. [26,31,32]. Future studies employing genetic perturbation, metabolic flux tracing, and primary human HSC models will be required to define the mechanistic hierarchy and translational generalizability of this pathway.

## 4. Materials and Methods

### 4.1. Animal Model

Male C57BL/6 mice (6–8 weeks old) were purchased from Guangdong Yaokang Biotechnology Co., Ltd. (Foshan, China). Animals were housed under specific pathogen-free conditions with a 12 h light/dark cycle at 20–25 °C and 40–60% relative humidity, with free access to standard chow and water. To avoid selection bias, 32 mice were randomly assigned to four groups (n = 8 each) using a cyclic allocation method: normal control, corn oil vehicle control, CCl_4_ model, and CCl_4_ + pirfenidone (PFD). A researcher not involved in subsequent experiments performed the allocation.

Liver fibrosis was induced by intraperitoneal injection of 20% (*v/v*) CCl_4_ (Macklin, Cat# C805325, Shanghai, China) diluted in corn oil (Aladdin, Cat# C116025, Shanghai, China) at 1 mL/kg body weight, twice weekly for 6 weeks. Mice in the CCl_4_ + PFD group additionally received PFD (400 mg/kg/day) by oral gavage on the five days without CCl_4_ injection each week. PFD was suspended in 0.5% sodium carboxymethyl cellulose (CMC-Na, Selleck, Cat# S6703, Houston, TX, USA) at 20 mg/mL, freshly prepared before each administration. The normal control and CCl_4_ model groups received an equal volume of 0.5% CMC-Na vehicle at the same time points.

During daily administration, the investigator monitored body weight, fur status, spontaneous activity, abdominal distension, and hunched posture. Humane endpoints were predefined as inability to eat or drink autonomously, severe ascites, persistent hunched posture/piloerection/lethargy, or visible tumors/infections. No mouse reached any endpoint. At the end of the experiment, mice were deeply anesthetized with isoflurane and euthanized by cervical dislocation, and liver tissues were harvested. All animal procedures were conducted at Institutional Ethics Committee of the Animal Center of Sun Yat-sen University Cancer Center and performed in accordance with institutional guidelines for animal care and use. (The ethics approval number is L102042024030D).

### 4.2. Cell Culture

The human hepatic stellate cell line LX-2 and the rat hepatic stellate cell line HSC-T6 were purchased from BDBIO (Shanghai, China). Cells were cultured in Dulbecco’s Modified Eagle Medium (DMEM; Gibco, Cat# 11965092, Waltham, MA, USA) supplemented with 10% fetal bovine serum (FBS; ExCell Bio, Cat# FSP500, Shanghai, China) and 1% antibiotic-antimycotic solution (NCM, Cat# C100C5, Suzhou, China) at 37 °C in a humidified incubator containing 5% CO_2_. LX2 and HSC-T6 cells were grown in 6 cm dishes and passaged at ratios of 1:2 and 1:3. All experiments were performed on cells between passages 5 and 15 after thawing. Cells in logarithmic growth phase were used for all experiments.

### 4.3. Reagents and Treatments

Recombinant human TGF-β1(Cat#100-21C) and TGF-β2(Cat#100-35B) were obtained from PeproTech (Cranbury, NJ, USA), while the rat isoforms(Cat# HY-P70648, HY-P70649) were purchased from MedChemExpress (MCE, Belleville, NJ, USA). Cells were treated with TGF-β1 or TGF-β2 at 10 ng/mL.

Four fatty acids were used: oleic acid (OA), palmitic acid (PA), linoleic acid (LA), and α-linolenic acid (ALA). OA and PA were obtained in both BSA-conjugated and DMSO-dissolved forms, while LA and ALA were only available in DMSO. The BSA-conjugated forms of OA and PA were purchased from Meilunbio (Cat# PWL220 for OA, Cat# PWL221 for PA; Dalian, China), and the DMSO-dissolved forms of all fatty acids were purchased from Sigma-Aldrich (Cat# O1008 for OA, Cat# P0500 for PA, Cat# L1376 for LA, Cat# L2376 for ALA; St. Louis, MO, USA). For fatty acid uptake assays, cells were treated with 200 μM of the indicated fatty acids. For all other experiments, including colony formation, Western blotting, immunofluorescence, and fatty acid production assays, a concentration of 25 μM was used. Pilot experiments confirmed that BSA- and DMSO-conjugated OA similarly promoted HSCs proliferation, whereas the corresponding vehicles had no effect. Given its physiological relevance and common use, BSA-conjugated OA was selected for all subsequent experiments.

Pirfenidone (PFD) was purchased from TargetMol (Cat# T2386, Boston, MA, USA). PFD was suspended in 0.5% sodium carboxymethyl cellulose (CMC-Na, Selleck, Cat# S6703, Houston, TX, USA) for in vivo administration. For in vitro use, PFD was dissolved in DMSO and diluted in culture medium to a final concentration of 250 μg/mL, with the final DMSO concentration kept below 0.1%.

### 4.4. Colony Formation Assay

Colony formation assays were performed using 12-well plates (approximately 3.8 cm^2^ bottom area per well). LX-2 and HSC-T6 cells were seeded at 500–1000 cells per well in complete medium containing the indicated treatments (TGF-β1, TGF-β2, OA, PA, LA, ALA, or PFD). The concentrations of reagents used are detailed in Methods Section 4.3. Cells were cultured for 10 days at 37 °C in 5% CO_2_, and the treatment-containing medium was refreshed every 3 days.

At the end of the incubation period, cells were gently washed with phosphate-buffered saline (PBS), fixed with 4% paraformaldehyde for 15–30 min at room temperature, and stained with 0.1% crystal violet for 20–30 min. After washing and air-drying, plates were photographed and colonies were counted. Colonies containing ≥50 cells were counted. For quantification, each treatment was analyzed in three independent biological replicates.

### 4.5. BODIPY 493/503 Staining and Intracellular Lipid Quantification

BODIPY staining was performed using a commercial kit (Beyotime, Cat# C2053S, Shanghai, China). All steps—including dye concentration, incubation time, and temperature—followed the manufacturer’s instructions. After treatment, cells were fixed with 4% paraformaldehyde for 10–15 min, incubated with BODIPY 493/503 working solution for 20–30 min in the dark, and counterstained with DAPI. Images were acquired using the same exposure settings within each experiment.

For experiments evaluating TGF-β-associated intracellular lipid accumulation, cells were serum-starved for 24 h and then stimulated with TGF-β1 or TGF-β2 (10 ng/mL) for 48 h. For experiments evaluating OA-associated intracellular lipid accumulation, cells were pretreated with TGF-β1 or TGF-β2 (10 ng/mL) for 24 h and then incubated with OA (200 μM) for the final 4 h in the continued presence of the corresponding TGF-β stimulus.

For quantification, at least three random fields were captured per sample by an experimenter blinded to group allocation. Mean fluorescence intensity per single cell was calculated as total BODIPY intensity per field divided by the number of nuclei (DAPI) in that field, using ImageJ 1.54f. Three independent replicate experiments were performed.

### 4.6. Oil Red O Staining

For cultured cells, treated cells were washed once with PBS, fixed with 4% paraformaldehyde for 10 min at room temperature, and stained with Oil Red O working solution (Beyotime, Cat# C0158S, Shanghai, China) for 1 h at room temperature. After removal of excess stain, cells were washed and imaged under a light microscope. For each group, multiple random fields were captured, and three fields were randomly selected for quantification of lipid droplet number per cell by a blinded observer. Data are presented as the mean ± SEM from a single experiment. The experiment was repeated three times with similar results.

For frozen liver sections, slides were equilibrated to room temperature, stained with Oil Red O working solution for 15 min, counterstained with hematoxylin, and mounted for microscopic examination.

### 4.7. Western Blotting

Cells were lysed in 1× RIPA buffer supplemented with protease inhibitor cocktail (TargetMol, Cat# C0001, Boston, MA, USA). Lysates were sonicated, and protein concentration was determined using a BCA protein assay kit (Thermo Scientific, Cat# 23225, Waltham, MA, USA). A total of 20 µg of protein per lane was denatured in loading buffer, separated by SDS-PAGE, and transferred onto PVDF membranes (Merck Millipore, Cat# IPVH00010, Burlington, VT, USA). Electrophoresis was run at 80 V for the stacking gel and 120 V for the resolving gel for 70–90 min. Transfer was performed by wet transfer at a constant current of 200 mA, with transfer time calculated as target protein molecular weight (kDa) plus 30 min. Membranes were blocked with 5% non-fat milk in TBST for 1–2 h at room temperature and incubated overnight at 4 °C with primary antibodies. After washing, membranes were incubated with HRP-conjugated secondary antibodies for 1–2 h at room temperature and visualized using enhanced chemiluminescence substrate (Bio-Rad, Cat# 1705061, Hercules, CA, USA).

Primary antibodies used were as follows: anti-phospho-SMAD2/3 (CST, Cat# 8828T, Danvers, MA, USA), anti-total SMAD2/3 (CST, Cat# 8685T, Danvers, MA, USA), anti-FASN (CST, Cat# 3180T, Danvers, MA, USA), anti-phospho-AKT (beyotime, Cat# AA329, Shanghai, China), anti-total AKT (beyotime, Cat# AA326, Shanghai, China), anti-phospho-ERK (proteintech, Cat# 80031-1-RR, Rosemont, IL, USA), anti-total ERK (proteintech, Cat# 66192-1-Ig, Rosemont, IL, USA), anti-phospho-p70 S6K (CST, Cat# 9234, Danvers, MA, USA), anti-total p70 S6K (CST, Cat# 9202, Danvers, MA, USA), anti-SREBP1 (Santa Cruz, Cat# sc-13551, Dallas, TX, USA) and appropriate loading controls such as GAPDH (proteintech, Cat# 60004-1-Ig, Rosemont, IL, USA). GAPDH at 1:5000, secondary antibodies at 1:5000, and all other primary antibodies at 1:1000. Protein band intensities were normalized to GAPDH. All experiments were performed in triplicate (n = 3).

### 4.8. Immunofluorescence Staining

Cells grown on confocal dishes were fixed with 4% paraformaldehyde after the indicated treatments, permeabilized with 0.1% Triton X-100 (Beyotime, Cat# P0096, Shanghai, China), and blocked with 5% bovine serum albumin. Cells were then incubated with primary antibodies overnight at 4 °C, followed by incubation with fluorescence-conjugated secondary antibodies for 1 h at room temperature in the dark. The primary antibodies used were anti-SREBP1 (Santa Cruz, Cat# sc-13551, Dallas, TX, USA) at 1:50, anti-FASN (CST, Cat# 3180T, Danvers, MA, USA) at 1:50. Nuclei were counterstained with DAPI, and images were acquired using a laser scanning confocal microscope under identical settings within each experiment.

### 4.9. Immunohistochemistry

Paraffin-embedded liver sections were deparaffinized, rehydrated, and subjected to antigen retrieval in sodium citrate buffer (pH 6.0) at 96 °C for 10 min, then cooled to room temperature. After blocking, sections were incubated with primary antibodies overnight at 4 °C: α-SMA (proteintech, Cat# 67735-1-Ig, Rosemont, IL, USA) at 1:1000, SREBP1 (Santa Cruz, Cat# sc-13551, Dallas, TX, USA) at 1:100, FASN at 1:250 (CST, Cat# 3180T, Danvers, MA, USA) and TGF-β1 (Affinity Biosciences, Cat# AF1027, Cincinnati, OH, USA) at 1:250. Negative controls were performed without primary antibody incubation. Signals were visualized using DAB substrate, and nuclei were counterstained with hematoxylin. Images were acquired under a light microscope at the magnifications indicated in the figure legends. The detailed scoring method for IHC staining is described in Section 4.11.

### 4.10. Multiplex Immunofluorescence/Multiplex Immunohistochemistry

Multiplex staining was performed using a sequential approach with a commercial kit (PANOVue, Cat# 10001100020, Beijing, China). Paraffin sections were first dewaxed and rehydrated, followed by antigen retrieval using a microwave in citrate buffer. The sections were then sequentially incubated with primary antibodies, corresponding secondary antibodies, and tyramide signal amplification (TSA) with fluorescent dyes. Between each round of staining, the sections underwent an additional antigen retrieval step to strip the bound antibody complexes. The primary antibodies were applied sequentially in the order: α-SMA, followed by SREBP1, and finally FASN. Antibody concentrations are detailed in Section 4.9. This strategy enabled multiplex detection of α-SMA, SREBP1, and FASN on the same section. Nuclei were counterstained with DAPI. To verify antibody specificity under multiplex conditions, multiplex staining results were compared with single staining, and signal localization and intensity were consistent between the two.

### 4.11. Histological Analysis and IHC Scoring

Liver tissue morphology was evaluated by hematoxylin and eosin (H&E) staining. For each staining indicator, one tissue section per animal was taken from eight animals per group. From each section, two fields of view were randomly captured. From the pooled fields (16 fields per group), six fields were randomly selected for quantification. All pictures were taken independently by two researchers blinded to group allocation.

For semiquantitative analysis, staining was evaluated using an integrated score based on staining intensity and positive area percentage. Staining intensity was scored manually on a scale of 0 to 3 (0, negative; 1, weak; 2, moderate; 3, strong), and positive area percentage was scored on a scale of 1 to 4 (1, 0–25%; 2, 26–50%; 3, 51–75%; 4, 76–100%). The final integrated score was calculated by multiplying the intensity score by the area score. Scoring was performed independently by two researchers blinded to group allocation, and the final score was the average of the two scores.

### 4.12. Statistical Analysis

A post hoc power analysis was performed using G*Power 3.1 based on the α-SMA staining results from the in vivo experiment.

All experiments were independently repeated three times. Normality was assessed using the Shapiro-Wilk test, and homogeneity of variances using Levene’s test. Data meeting normality and equal variance assumptions were analyzed by one-way ANOVA followed by Tukey’s HSD post hoc test. Data not meeting these assumptions were analyzed by the Kruskal-Wallis test followed by Dunn’s post hoc test. Two-way ANOVA with Šídák’s post hoc test was used for comparisons between PFD-treated and untreated groups. For comparisons between two groups, an unpaired two-tailed Student’s *t*-test was used. A *p*-value < 0.05 was considered statistically significant. All statistical analyses were performed using GraphPad Prism version 9.5.

## Data Availability

The raw data supporting the conclusions of this article will be made available by the authors on request.

## Supplementary Materials

The following supporting information can be downloaded at: https://www.mdpi.com/article/10.3390/ijms27094061/s1.

## Abbreviations

The following abbreviations are used in this manuscript:

HSC	hepatic stellate cell
NAFLD	non-alcoholic fatty liver disease
TGF-β	transforming growth factor-β
SREBP1	sterol regulatory element-binding protein 1
FASN	fatty acid synthase
PFD	pirfenidone
OA	oleic acid
LA	linoleic acid
ALA	α-linolenic acid
PA	palmitic acid
CCl_4_	carbon tetrachloride
DMEM	Dulbecco’s Modified Eagle Medium
FBS	fetal bovine serum
PBS	phosphate-buffered saline
DAPI	4′,6-diamidino-2-phenylindole
SDS-PAGE	sodium dodecyl sulfate–polyacrylamide gel electrophoresis
PVDF	polyvinylidene fluoride
HRP	horseradish peroxidase
BCA	bicinchoninic acid
TBST	Tris-buffered saline with Tween 20
TSA	tyramide signal amplification
H&E	hematoxylin and eosin
