# Supplementary material for: Pirfenidone Suppresses Liver Fibrosis Through Inhibition of TGF-β-Associated Lipid Metabolic Remodeling in Hepatic Stellate Cells
Source: Int J Mol Sci. 2026 Apr 30;27(9):4061. doi: 10.3390/ijms27094061 (PMC13164092; doi:10.3390/ijms27094061)
Supplement: Supplementary file 1 [file ijms-27-04061-s001.zip › ijms-4248301-supplementary.pdf]

Pirfenidone Suppresses Liver Fibrosis through Inhibition of TGF-β-Associated Lipid Metabolic Remodeling in Hepatic Stellate Cells

Supplementary Materials

Yuelu Lan [1], Sijia Li [1], Shuangli Zhu [1], Can Pan [1], Kai Fu [1], Xueping Wang [1], Liwu Fu [1*], and Fang Wang [1*]

[1] State Key Laboratory of Oncology in South China, Guangdong Provincial Clinical Research Center for Cancer, Sun Yat-sen University Cancer Center

* Correspondence:

Liwu Fu, E-mail: fulw@mail.sysu.edu.cn; Tel.: +86-20-873-431-63; Fax: +86-20-873-431-70;

Fang Wang, E-mail: wangf@sysucc.org.cn; Tel.: +86-20-873-427-13; Fax: +86-20-873-431-70;

1. Supplementary Figures

Figure S1.

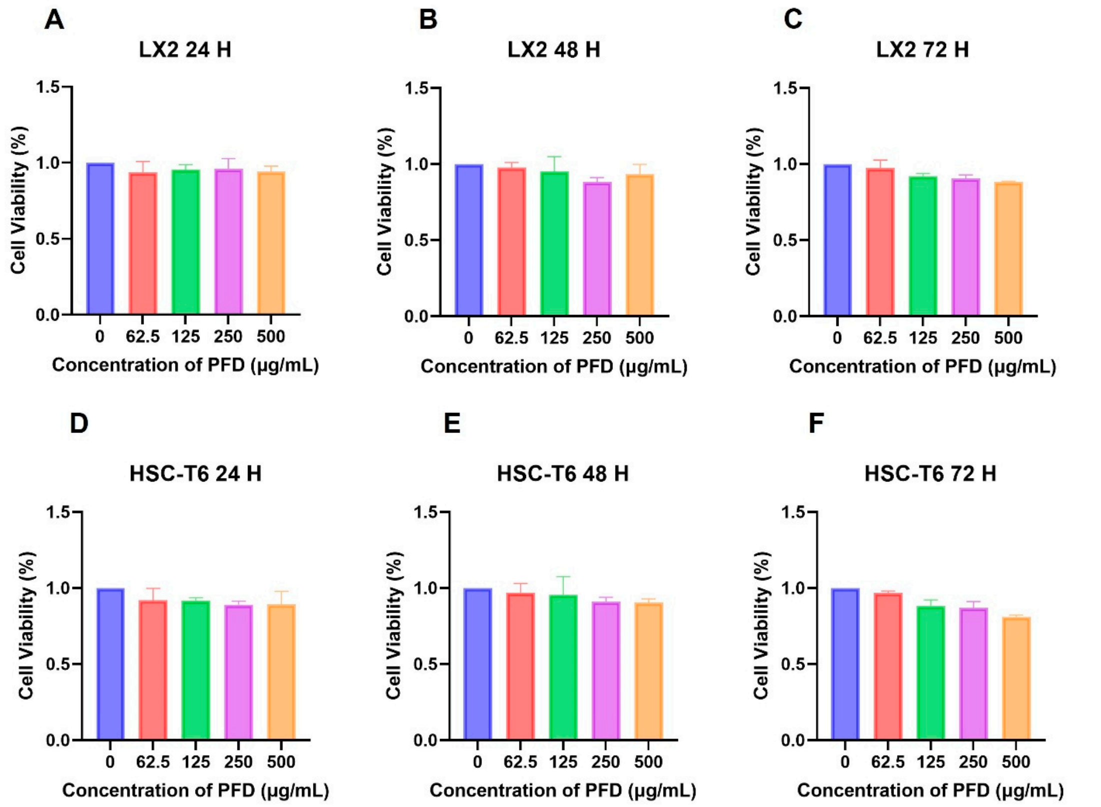

Figure S1. Effect of pirfenidone on cell viability.

(A) LX2 cells were treated with indicated concentrations of pirfenidone (0, 62.5, 125, 250, 500 μg/mL) for 24, 48, or 72 h, and cell viability was assessed by MTT assay. (B) HSC-T6 cells were treated with indicated concentrations of pirfenidone (0, 62.5, 125, 250, 500 μg/mL) for 24, 48, or 72 h, and cell viability was assessed by MTT assay. Data are presented as the mean ± SEM.

Figure S2.

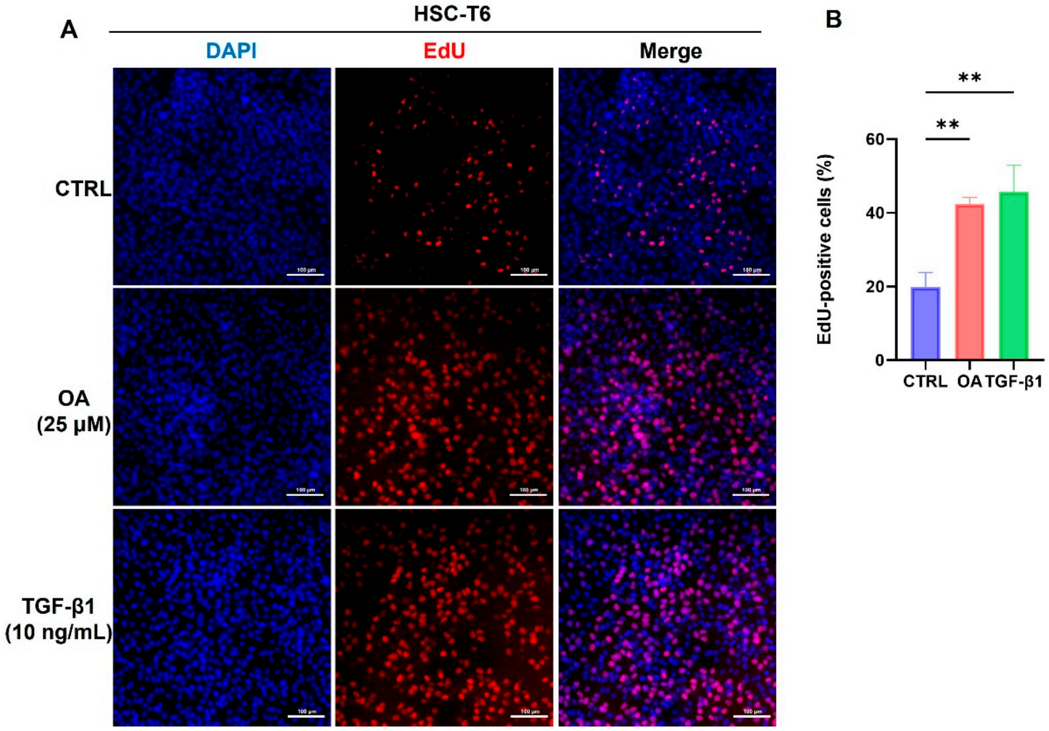

Figure S2. EdU assay for cell proliferation assessment.

(A) Images of EdU staining in HSC-T6 cells treated with 25 μM OA or 10 ng/mL TGF-β1 for 48 h, followed by incubation with 10 μM EdU for 2 h. EdU-positive cells (red) and nuclei (DAPI, blue) are shown. Images were acquired under randomized and blinded conditions. (B) Quantification of EdU-positive cells (%). Quantification was performed from randomly selected fields under blinded conditions. Significance was determined by one-way ANOVA with Tukey's HSD post hoc test. Data are presented as the mean ± SEM. *$p < 0.05$, **$p < 0.01$ vs. control group.

Figure S3.

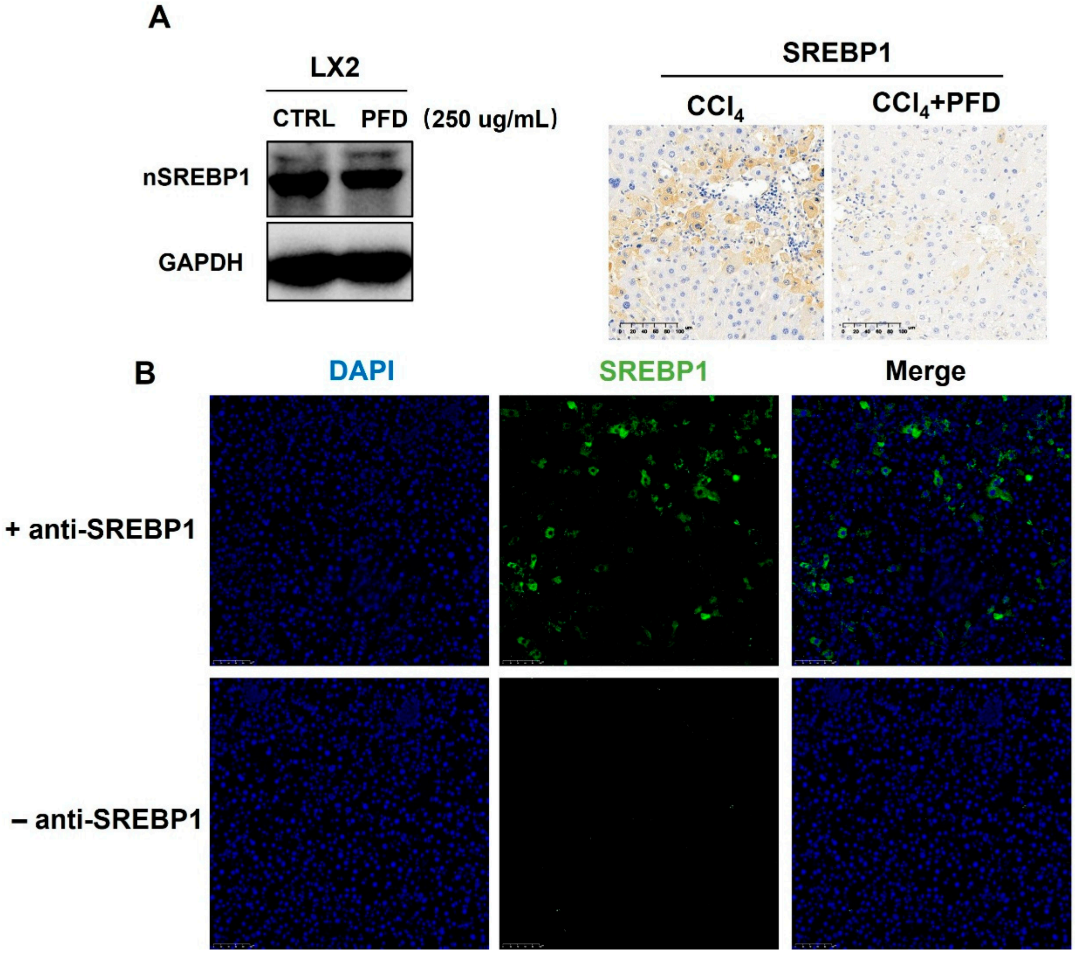

Figure S3. Validation of SREBP1 antibody specificity.

(A) SREBP1 protein expression detected by Western blot in LX2 cells treated with 250 μg/mL PFD for 48 h (left), and immunohistochemistry staining of SREBP1 in liver tissues from CCl_4-treated mice with or without PFD treatment (right). (B) Multiplex immunohistochemistry single staining for SREBP1 with 570 nm dye, showing specific signal in the presence of SREBP1 antibody compared to no primary antibody control.

2. Supplementary Methods

2.1 MTT cytotoxicity assay

LX2 cells (3,000 cells/well) and HSC-T6 cells (1,500 cells/well) were seeded in 96-well plates and treated with indicated concentrations of pirfenidone (0, 62.5, 125, 250, 500 μg/mL) for 24, 48, or 72 h. MTT solution (MP Biomedicals, Cat# 0210222701) was added and incubated for 4 h at 37 °C. Formazan crystals were dissolved in DMSO, and absorbance was measured at 570 nm with background correction at 630 nm. Cell viability was normalized to untreated controls.

2.2 EdU proliferation assay

EdU incorporation was assessed using the EdU Kit (Beyotime, Cat# C0071S) following the manufacturer's instructions. Cells were incubated with EdU (10 μM) for 2 h at 37 °C, then fixed, permeabilized, and stained with Click reaction mixture. Nuclei were counterstained with DAPI. Images were acquired from randomly selected fields under blinded conditions. Quantification of EdU-positive cells (%) was performed from randomly selected fields under blinded conditions. Data are presented as the mean ± SEM. Significance was determined by one-way ANOVA with Tukey's HSD post hoc test. *$p < 0.05$, **$p < 0.01$ vs. control group..
